# To identify errors in prescribing practice in an inpatient psychiatric unit and improve compliance with Mental Health Commission of Ireland regulations relating to prescribing

**DOI:** 10.1192/bjo.2021.102

**Published:** 2021-06-18

**Authors:** Kate Corrigan, Rebecca Conlan-Trant, Shannon Cleary

**Affiliations:** Department of Psychiatry, Tallaght University Hospital

## Abstract

**Aims:**

Prescribing errors can lead to patient harm and are a patient safety issue. In 2019 the Acute Psychiatric Unit in Tallaght Hospital was identified by the Mental Health Commission of Ireland as non-compliant with regulation 23 of the Mental Health Act pertaining to the Ordering, Prescribing, Storing and Administration of Medication. Compliance with regulation 23 is a mandatory condition for the registration of the Unit as an Approved Centre to provide treatment for mental illness in Ireland. Regular auditing was performed to identify areas of non-compliance in prescribing practices and where identified to improve upon these practices per Mental Health Commission standards.

**Method:**

A cross sectional review of 14–18 medication Kardexes was completed monthly from August – December 2020. Kardexes were audited against 20 standards set by the Mental Health Commission. An electronic audit tool was used to collect data. Medical teams were informed of any incidences of non-compliance. Education sessions delivered by both medical staff and the ward pharmacist were provided to junior doctors and consultants regarding the Mental Health Commission regulations for prescribing. We developed information leaflets that were placed at the front of Kardex folders highlighting key areas where errors were regularly made. Monthly staff emails were sent reminding prescribers of the importance of adhering to guidelines and updating them on the most recent audit results.

**Result:**

Improvements were noted in all aspects of prescribing over the five-month period. Prescriptions of non-proprietary medication improved from 40% of Kardexes to 87% over the five-month period. Recording of prescriber medical registration number improved from 80% to 87% of Kardexes. Documentation of the dates of initiation and discontinuation of a medication improved from 40% to 67%. The use of appropriate patient identifiers on Kardexes improved from 93% to 100%.

**Conclusion:**

Targeting staff across multiple domains including emails, information leaflets and education sessions resulted in consistent improvements in medication prescribing. The Mental Health Commission has since inspected the Acute Psychiatric Unit in Tallaght Hospital in 2020 and deemed it fully complaint with regulation 23 pertaining to medication prescribing.

